# A pan-cancer transcriptome analysis identifies replication fork and innate immunity genes as modifiers of response to the CHK1 inhibitor prexasertib

**DOI:** 10.18632/oncotarget.27400

**Published:** 2020-01-21

**Authors:** Wayne D. Blosser, Jack A. Dempsey, Ann M. McNulty, Xi Rao, Philip J. Ebert, Caitlin D. Lowery, Philip W. Iversen, Yue Wang Webster, Gregory P. Donoho, Xueqian Gong, Farhana F. Merzoug, Sean Buchanan, Karsten Boehnke, Chunping Yu, Xin Tian You, Richard P. Beckmann, Wenjuan Wu, Samuel C. McNeely, Aimee Bence Lin, Ricardo Martinez

**Affiliations:** ^1^ Eli Lilly and Company, Indianapolis, IN, USA; ^2^ Eli Lilly and Company, New York, NY, USA; ^3^ Eli Lilly and Company, Shanghai, China

**Keywords:** CHK1, prexasertib, replication stress, replication fork, innate immunity

## Abstract

The combined influence of oncogenic drivers, genomic instability, and/or DNA damage repair deficiencies increases replication stress in cancer. Cells with high replication stress rely on the upregulation of checkpoints like those governed by CHK1 for survival. Previous studies of the CHK1 inhibitor prexasertib demonstrated activity across multiple cancer types. Therefore, we sought to (1) identify markers of prexasertib sensitivity and (2) define the molecular mechanism(s) of intrinsic and acquired resistance using preclinical models representing multiple tumor types. Our findings indicate that while cyclin E dysregulation is a driving mechanism of prexasertib response, biomarkers associated with this aberration lack sufficient predictive power to render them clinically actionable for patient selection. Transcriptome analysis of a pan-cancer cell line panel and *in vivo* models revealed an association between expression of E2F target genes and prexasertib sensitivity and identified innate immunity genes associated with prexasertib resistance. Functional RNAi studies supported a causal role of replication fork components as modulators of prexasertib response. Mechanisms that protect cells from oncogene-induced replication stress may safeguard tumors from such stress induced by a CHK1 inhibitor, resulting in acquired drug resistance. Furthermore, resistance to prexasertib may be shaped by innate immunity.

## INTRODUCTION

Replication stress (RS) is biochemically defined as the uncoupling of helicase-driven unwinding and the advancement of DNA polymerases at the DNA replication fork. Most cancers are under RS as a consequence of the oncogenes that drive continuous cell proliferation and promote genomic instability [1]. Cancers survive this oncogene-induced replication stress (OIRS) by upregulating multiple stress pathways including ataxia-telangiectasia-mutated-and-Rad3-related kinase - checkpoint kinase 1 (ATR-CHK1) signaling. Activation of CHK1 by ATR in response to excess RS or DNA damage results in CHK1-mediated phosphorylation of the CDK2 phosphatase CDC25A, targeting it for proteolytic destruction. In the absence of CDC25A, the inhibitory phosphorylation of CDK2 at tyrosine residue 15 is maintained thereby pausing S phase to allow for DNA damage repair (DDR) and resolution of DNA replication conflicts [2, 3].

A key mechanism of OIRS involves excessive oncogene-induced CDK2-mediated replication origin firing resulting in depletion of nucleotide pools, fork stalling, double strand DNA breaks, and replication fork collapse [4]. ATR-CHK1 pathway blockade in systems with high RS via CHK1 inhibitors results in unscheduled DNA synthesis even in the presence of DNA damage; the bypass of the RS response checkpoint (also known as the intra-S checkpoint) results in DNA fragmentation, replication catastrophe, and cell death [5]. Tumor dependence on the ATR-CHK1 pathway to survive OIRS provides the rationale for the development and clinical investigation of small molecule kinase inhibitors targeting ATR (e. g. AZD6738), ATM (e. g. AZD1390), WEE1 (e. g. AZD1775) and CHK1, prexasertib being an example of the latter [6–11]. Prexasertib is a small molecule kinase inhibitor displaying high selectivity for CHK1 followed by CHK2 [5].

Extreme levels of OIRS could underlie, in principle, outlier response to prexasertib in certain patients treated with prexasertib monotherapy. Although our understanding of the causes and consequences of RS and its relevance to the ATR-CHK1 pathway has advanced considerably, these insights have not translated into the identification of biomarkers with the necessary predictive power for clinical implementation. A phase 1 clinical trial (NCT01115790) of single-agent prexasertib in head and neck squamous cell carcinoma (HNSCC) and squamous cell carcinoma of the anus (SCCA) identified loss-of-function (LOF) mutations in the E3 ubiquitin ligase *FBXW7* in patients with clinical benefit [12]. High cyclin E1 expression, as measured by immunohistochemistry, was linked to clinical benefit of prexasertib monotherapy in a heavily pre-treated high-grade serous ovarian cancer (HGSOC) patient population [13]. Interestingly, cyclin E1 over-expression has been linked to enhanced RS, with Jones *et al.* demonstrating that elevated levels of this cyclin could augment RS by promoting increased DNA replication, origin firing and impaired replication fork progression, and thus DNA damage [14]; however, the underlying mechanism remains to be fully elucidated.

We investigated the mechanistic underpinnings of prexasertib response in a variety of tumor types that were of interest to the clinical program, including squamous, pediatric (rhabdomyosarcoma), ovarian and triple-negative breast cancers. First, we explored a clinically informed hypothesis pointing to cyclin E dysregulation as a mechanism of prexasertib sensitivity. Second, we investigated a tumor-agnostic panel of preclinical models (cancer cell lines and *in vivo* models) for markers of response via multi-omic profiling. Finally, multiple carcinoma and sarcoma models of acquired resistance to prexasertib were established and molecularly characterized to gain insight into mechanism(s) associated with acquired resistance. Our observations highlight the challenge associated with identifying single, recurrent biomarkers with sufficient predictive power for clinical implementation. Notwithstanding these obstacles, the preclinical observations in this report advance our understanding of the molecular basis of response to prexasertib and shed light into mechanisms underlying intrinsic and acquired drug resistance. Importantly, transcriptional profiling emerges as a particularly informative approach in helping define the molecular context of response to prexasertib.

## RESULTS

### Cyclin E dysregulation is associated with enhanced sensitivity to prexasertib

**Figure 1:**
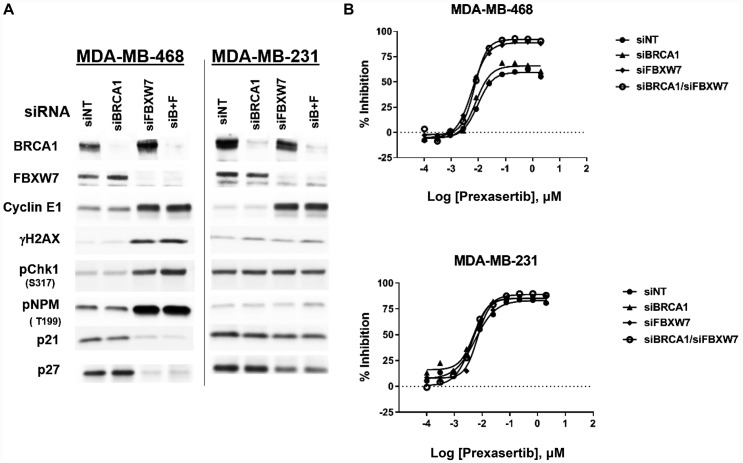
Effect of *FBXW7* knockdown on cyclin E1 levels and prexasertib sensitivity. Two triple-negative breast cancer tumor cell lines, MDA-MB-468 and MDA-MB-231, were exposed to siNT (non-target), si*FBXW7*, si*BRCA1*, or a combination of the two for 48 hours. (**A**) Effects on the siRNA target and CHK1 pathway-related proteins were assessed by western blot analysis. (**B**) Cell viability following prexasertib treatment of MDA-MB-468 or MDA-MB-231 cells exposed to NT or on-target siRNAs. Equal protein loaded in all lanes.

Previously, loss-of-function mutations in *BRCA1* and *FBXW7*, an E3 ubiquitin ligase that marks cyclin E for proteolytic destruction [15], were associated with clinical benefit in patients with HNSCC or SCCA [12]. We hypothesized that loss of *FBXW7* would lead to increased cyclin E1 and thus shift the equilibrium of CDK2 towards its active state, resulting in elevated basal level of RS and sensitivity to CHK1 inhibition. Given limitations on number and accessibility of well-characterized HPV+ HNSCC or SCCA cell lines, we used breast and ovarian cancer lines. *FBXW7* mutations and amplification of *CCNE1* (the gene that encodes cyclin E1) occur in these tumor types (*FBXW7* mutations: approximately 1.5% of breast and ovarian cancers; *CCNE1* amplification [> 4 copies]: 2.3% in breast cancer, 17.6% in ovarian cancer based on TCGA_B38 data). Relative to a non-target control (siNT), siRNA-mediated knockdown of *FBXW7* in the triple-negative breast cancer (TNBC) cell line MDA-MB-468 resulted in elevated cyclin E1, DNA damage as evidenced by increased γH2AX, and CHK1 pathway activation as measured by CHK1 phosphorylation at S317 (the ATR-mediated phosphorylation site), (Figure 1A). In addition, increased phosphorylation of the CDK2 substrate nucleophosmin [16] was observed, indicative of elevated CDK2 activity following *FBXW7* knockdown (Figure 1A). In contrast to MDA-MB-468, *FBXW7* knockdown did not increase, cyclin E1 levels, CHK1 activation, DNA damage, or RS in a second TNBC cell line, MDA-MB-231. Moreover, whereas MDA-MB-468 si*FBXW7* cells showed decreased levels of CDK2 negative regulators p21 and p27 with a concomitant elevation in pNPM, the levels of these proteins showed significant less reduction in MDA-MB-231 si*FBXW7* compared to MDA-MB-468 si*FBXW7* cells. This suggests the possibility that CDK2 activation was suppressed or not enhanced in MDA-MB-231 si*FBXW7* cells despite increased cyclin E1 relative to siNT cells (Figure 1A). Consistent with a link between CDK2 activation and drug response, *FBXW7* knockdown sensitized MDA-MB-468 but not MDA-MB-231 cells to the CHK1 inhibitor (Figure 1B). Interestingly, *BRCA1* knockdown was not associated with elevated RS or DNA damage (Figure 1A), nor did it sensitize to prexasertib response in a cell viability assay (Figure 1B).

### Across a pan-cancer cell line panel, the expression of E2F/G2M/SAC genes tracks with sensitivity while the expression of immune-related genes is associated with intrinsic resistance to prexasertib

**Figure 2:**
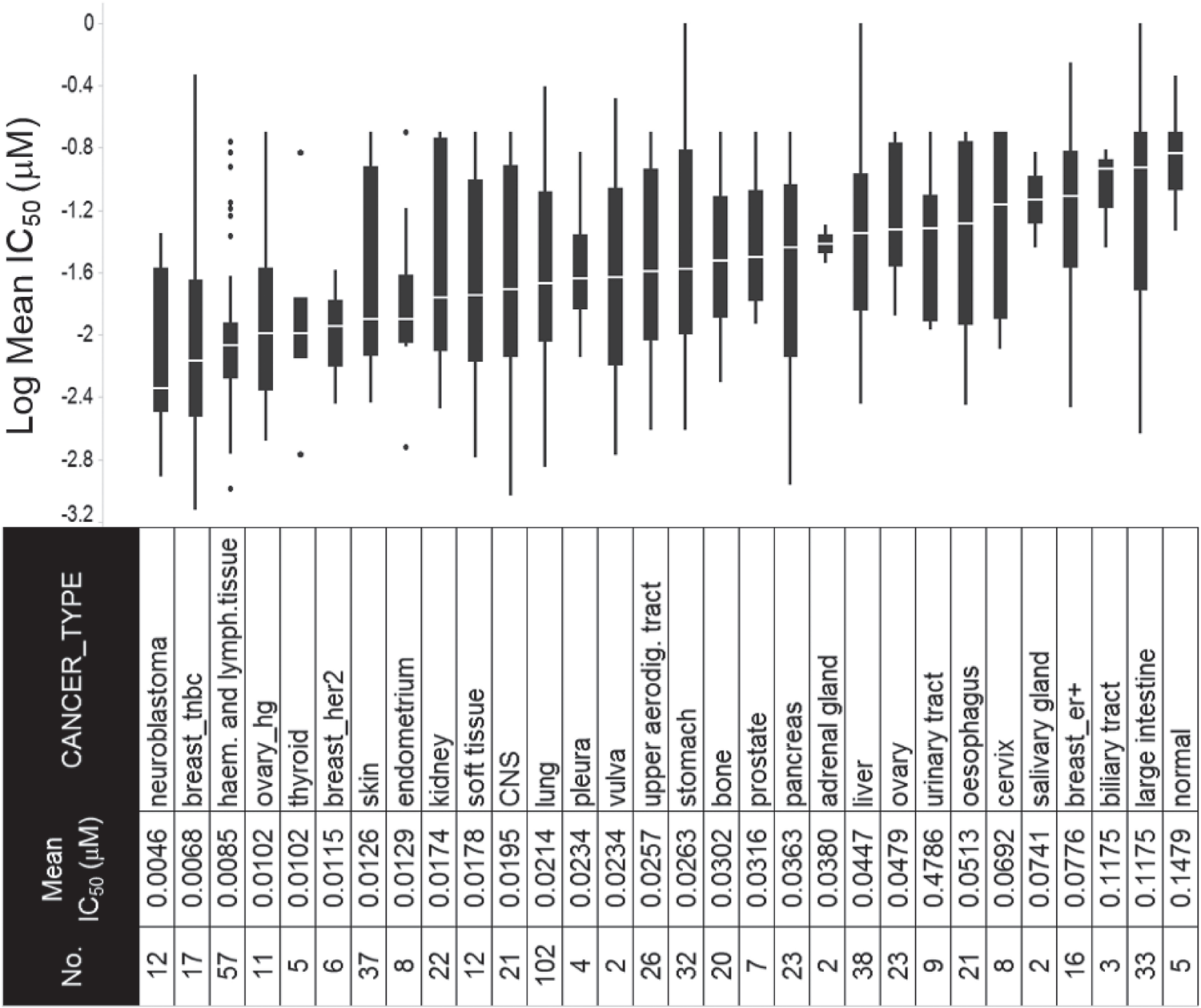
Response to prexasertib treatment across a pan-cancer cell line panel. The panel is composed of 572 well-characterized cancer cell lines representing 29 different adult and pediatric tumor types. Y-axis shows log of the mean of the absolute IC_50_ value from replicate measurements (see Supplementary Table 1); x-axis displays the cancer type, abbreviation, and corresponding number of cell lines tested.

In an effort to investigate underlying mechanisms of response to prexasertib in an unbiased manner, we used a tumor-agnostic approach using a previously described, molecularly well-characterized pan-cancer cell line panel consisting of 572 tumor lines comprising 29 cancer types (Figure 2, Supplementary Table 1) [17]. The geometric mean of the IC_50_ value was calculated from independent measurements (ranging from 1–18, depending on cell line). Response to prexasertib varied widely both between and within histologies (Figure 2, Supplementary Table 1). Tumor types that displayed the greatest sensitivity to prexasertib included neuroblastoma (NBL) previously reported in [18], TNBC, HER2^+^ breast cancer, and HGSOC. The opposite end of the prexasertib response spectrum included estrogen receptor-positive (ER^+^) breast, low-grade serous or other ovarian cancers, as well as colorectal, liver, and cervical cancers. Pre-malignant (e. g. MCF10A) and normal cell cultures were the least sensitive to prexasertib.

**Figure 3:**
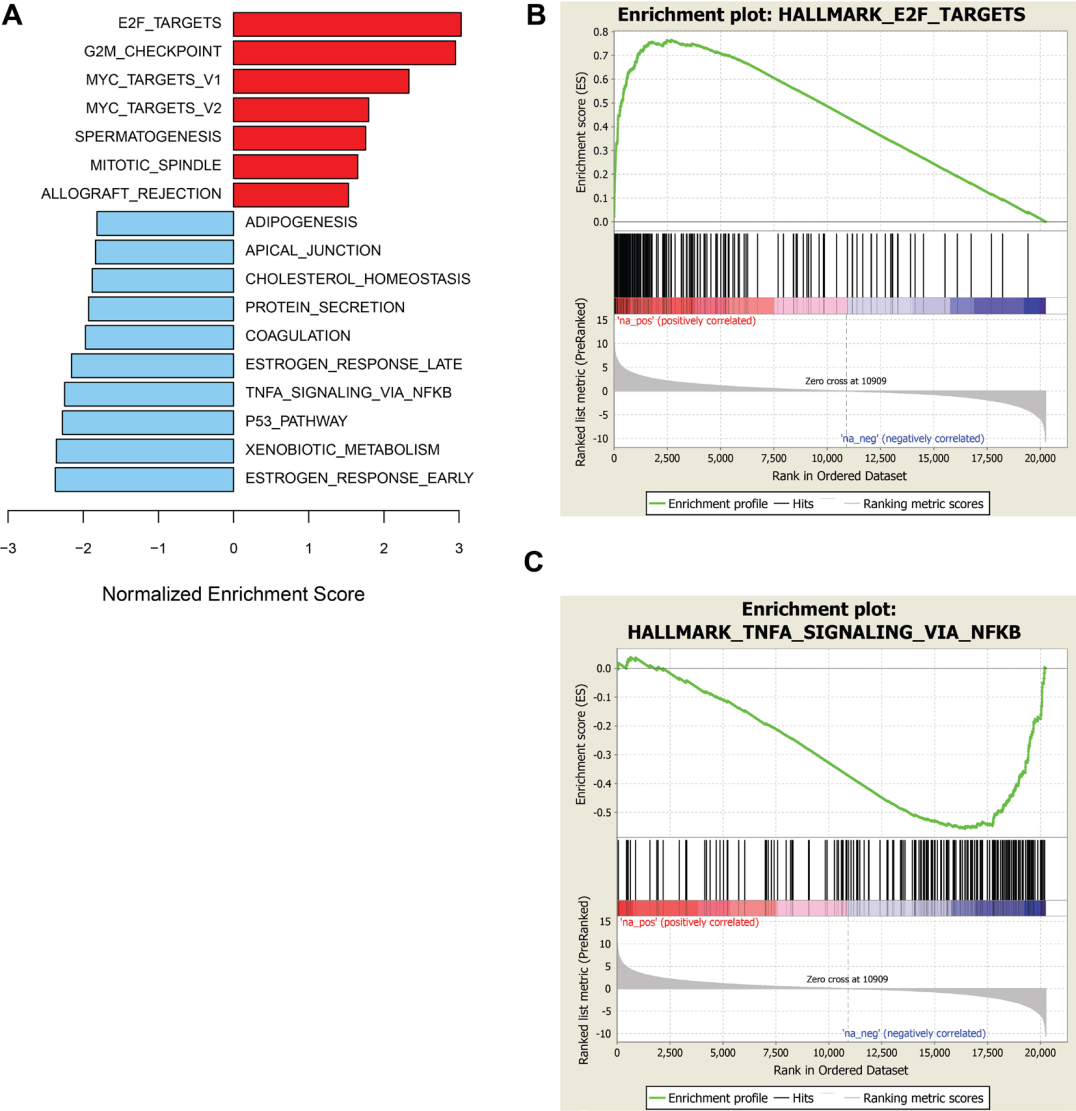
GSEA of genes associated with sensitivity or resistance to prexasertib in pan-cancer cell line panel. Bar plot of top hallmark gene sets associated with sensitivity (red) or resistance (blue) using FDR<0.05 (**A**) and representative enrichment plots highlighting top-scoring gene sets associated with prexasertib sensitivity (**B**) or intrinsic resistance (**C**).

**Figure 4:**
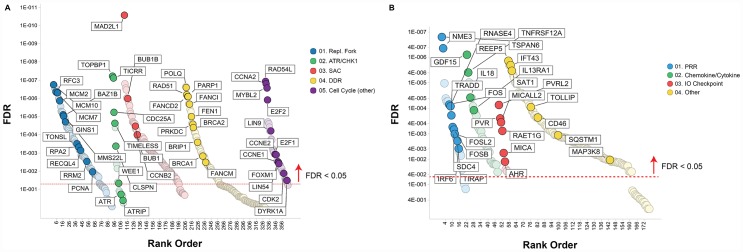
Pathways associated with prexasertib sensitivity (**A**) or resistance (**B**) across pan-cancer cell line panel. Association between gene expression from RNASeq data and drug response (geometric mean IC_50_) was calculated as described in the Materials and Methods section and plotted as a function of FDR (y-axis) and gene classification (x-axis) according to pathways and gene sets identified by GSEA.

To identify molecular traits tracking with prexasertib response across the pan-cancer cell line panel genetic variants, including mutations, insertion/deletions, frame-shift alterations, splice mutations, and copy number alterations derived from whole-exome analysis were investigated for association with response to prexasertib. None of the variants identified passed a statistical cutoff (FDR<0.05). In sharp contrast to the lack of statistically significant association between prexasertib response and genetic variants identified by exome analysis, gene set enrichment analysis (GSEA) of whole transcriptome data revealed transcriptional features that tracked with drug response. RNASeq analysis identified 2,974 genes which significantly correlated with sensitivity to prexasertib (FDR<0.05) (Supplementary Table 2). To establish whether these genes fall into specific biological pathways, GSEA was conducted as previously described [19]. The most significantly associated hallmarks with sensitivity to prexasertib included E2F transcription factor targets, G2M checkpoint, MYC targets, mitotic spindle assembly checkpoint (SAC), and DDR (henceforth called the E2F/G2M/SAC signature) (Figure 3A, 3B). Inspection of the genes making up this signature identified several CHK1 mechanism-proximal components involved in the replication fork and ATR-CHK1 pathway activation, many of which are targets of E2F transcription factors (Figure 4A). Importantly, genes encoding proteins implicated in active and stressed forks that emerged from proteomic (IPOND) studies (e. g. BAZ1B, TONSL) [20, 21] displayed strong association with prexasertib sensitivity. Consistent with the observed E2F signature shown in Figure 3A and 3B, E2F transcription factors (*E2F1, E2F2*) and genes encoding CDK2-binding cyclins (*CCNE1, CCNE2*), which are required for active replication origin firing [22], were associated with sensitivity to prexasertib. Also noted was a distinct enrichment for association of expression of components of the DREAM pathway (*CCNA2, RAD54L, MYBL2, FOXM1, LIN54*) with prexasertib sensitivity.

The transcriptomic dataset from the pan-cancer cell line panel was also investigated by GSEA using 2,718 genes identified by RNASeq that significantly associated with intrinsic resistance to prexasertib (FDR<0.05) (Supplementary Table 3). GSEA identified estrogen response, xenobiotic metabolism, the p53 signaling pathway and tumor necrosis factor alpha signaling, as the most strongly associated gene sets with resistance (Figure 3A, 3C). Close inspection of the resistance-associated genes revealed components of innate immunity, including stimulator of interferon (STING) pathway genes such as pattern recognition receptors (PRR), chemokines and cytokines, immune checkpoints, and other interferon-induced genes (Figure 4B).

### In a sarcoma/neuroblastoma xenograft cohort, the expression of E2F/G2M/SAC genes tracks with sensitivity while the expression of immunity genes is associated with intrinsic resistance to prexasertib

A previous study from our laboratory published by Lowery *et al.* [23] demonstrated that alveolar rhabdomyosarcoma (aRMS) and neuroblastoma (NBL) xenograft models were the most sensitive to single-agent prexasertib when compared to other pediatric tumor types (including osteosarcoma [OS], Ewing’s sarcoma [ES], and non-sarcoma soft tissue tumors) as well as adult sarcoma (namely, leiomyosarcoma [LMS] and liposarcoma [LPS]). Therefore, we conducted a meta-analysis combining exome and transcriptome profiling generated using xenograft models from this previous study with additional models as part of our current study. As expected, copy number amplification of *MYCN* was detected in the KELLY and IMR-32 neuroblastoma models as were characteristic gene rearrangements in alveolar rhabdomyosarcoma (aRMS) (PAX3-FOXO1) and Ewing sarcoma (EWS-FLT1) models (Supplementary Table 4). *MYCN* mRNA expression appeared to track with evidence of genomic amplification in NBL. Furthermore, *MYCN,* a known target of the PAX3-FOXO1 fusion transcription factor [24] showed elevated expression levels in the two aRMS models, though not as high as in the *MYCN*-amplified NBL models. Prexasertib response appeared to track with the presence of MYC/MYCN amplification and PAX3-FOXO1 rearrangements (Supplementary Table 4).

**Figure 5:**
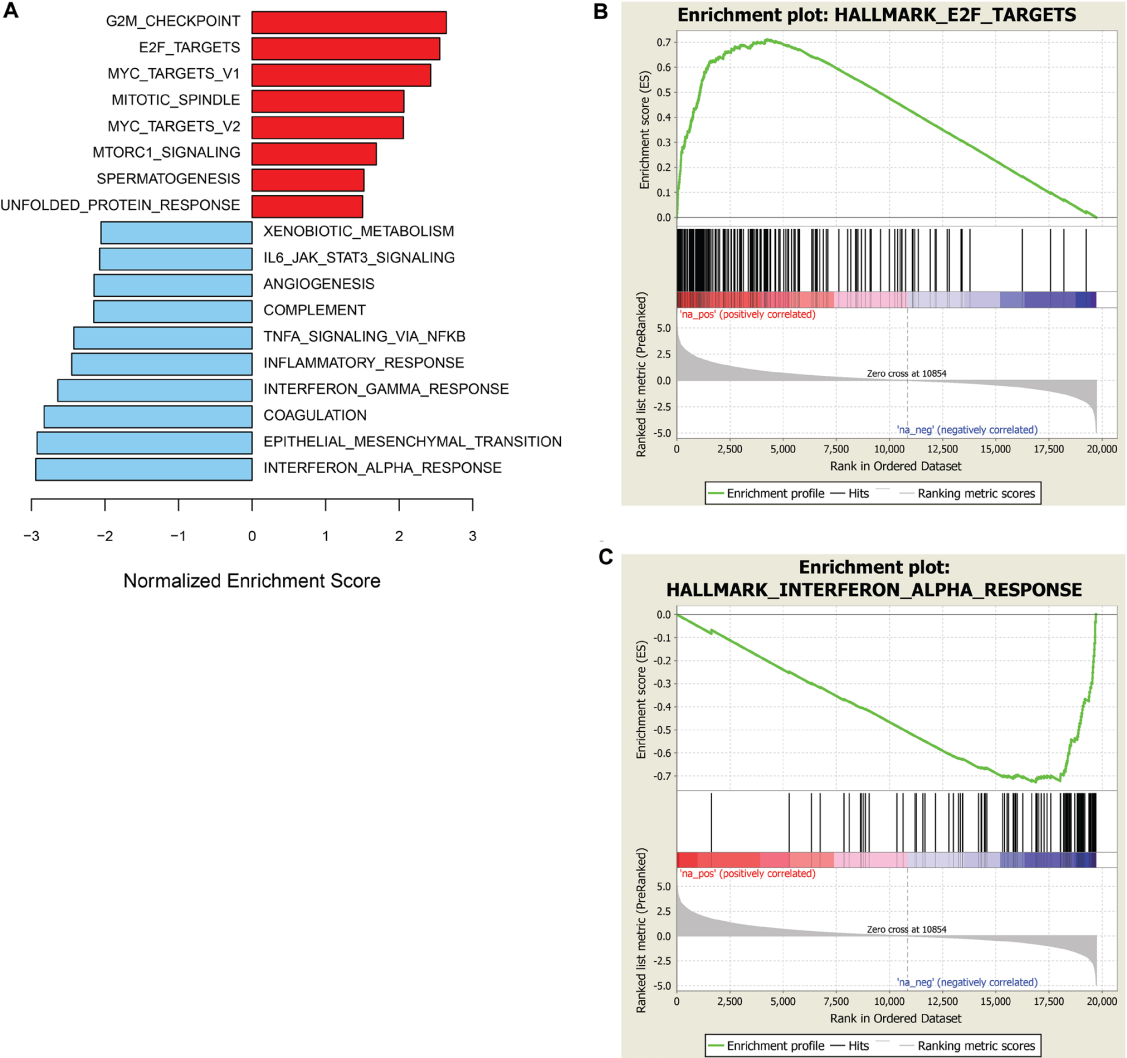
GSEA of genes associated with sensitivity or resistance to prexasertib in the sarcoma and neuroblastoma xenograft cohort. Bar plot of top hallmark gene sets associated with sensitivity (red) or resistance (blue) using FDR<0.05 (**A**) and representative enrichment plots highlighting top scoring gene sets associated with prexasertib sensitivity (**B**) or intrinsic resistance (**C**).

GSEA of the RNASeq data from the sarcoma and neuroblastoma xenograft models revealed a composite of gene sets associated with prexasertib sensitivity or resistance with a striking resemblance to those encountered in the pan-cancer cell line panel shown in Figure 3. Included in these gene sets were E2F target genes corresponding to sensitivity and IFN-alpha and -gamma-related genes as resistance signals (Figure 5A–5C, Supplementary Tables 5, 6).

### Acquired resistance to prexasertib in NCI-H520 is associated with lower RS or DNA damage and upregulated expression of the E2F/G2M/SAC signature

**Figure 6:**
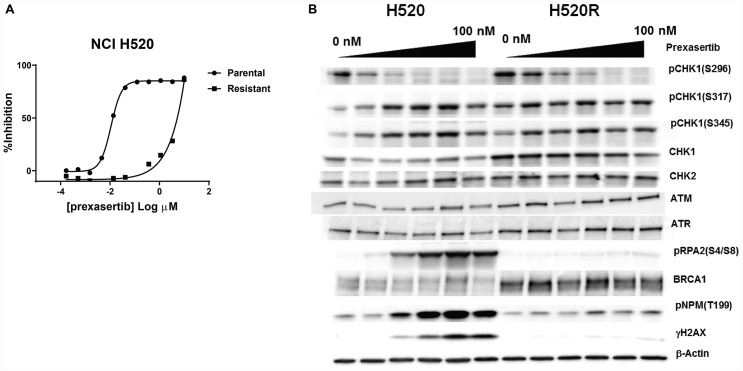
Pharmacological and molecular characterization of NCI-H520R cells. (**A**) Viability of parental NCI-H520 and its prexasertib-resistant version (NCI-H520R) was evaluated following prexasertib treatment for 96 hours. (**B**) Analysis of selected cell cycle-related proteins extracted from DMSO- or prexasertib-treated cultures for 24 hours. Drug concentrations used: 0, 6.25, 12.50, 25, 50, and 100 nM.

While the search for baseline predictive biomarkers of response across large datasets can generate hypotheses, evaluating the mechanistic contribution and causal role of such markers requires genetic and/or pharmacological approaches, ideally setup in an isogenic context to probe causality. Therefore, we generated a prexasertib-resistant line from the squamous lung adenocarcinoma NCI-H520 cancer cell line and used the parental (NCI-H520) and resistant (NCI-H520R) lines as a convenient isogenic pair to investigate further. The choice of a tumor line derived from a squamous cancer was dictated by the earliest signals of clinical activity of the prexasertib molecule in squamous tumors [25]. Evaluation of response to prexasertib for the parental and resistant tumor lines in a cell viability assay revealed a >400-fold shift in IC_50_ between NCI-H520 and NCI-H520R cells (0.011 μM versus 4.7 μM, respectively) (Figure 6A). Western blot analysis of NCI-H520 and NCI-H520R whole cell lysates revealed only subtle differences at baseline, with higher levels of BRCA1 in NCI-H520R compared to parental cells being one of the more noteworthy distinctions (Figure 6B). Markers of RS and DNA damage, pRPA2 and γH2AX, respectively, were observed in response to drug treatment in the parental cells but were nearly absent in drug-treated drug-resistant cells. Importantly, decreasing auto-phosphorylation of CHK1 at S296 was observed in drug-treated cells for both parental and resistant tumor lines, indicative that prexasertib was still inhibiting CHK1 kinase activity (Figure 6B). This observation suggests that drug resistance is not a consequence of poor drug exposure or lack of target inhibition. Moreover, phosphorylation of NPM at T199 was lower in drug-treated resistant cells when compared to the parental, indicating that CDK2 activity may be reduced in prexasertib-resistant cells potentially accounting for the strong attenuation in markers of RS (pRPA2) and DNA damage (γH2AX) in drug-treated cultures (Figure 6B).

We extended the search for prexasertib resistance markers by using Digiwest proteomic technology to provide a quantitative measure of nearly 300 proteins in NCI-H520 and NCI-H520R cells [26]. A distinct difference in markers of epithelial-mesenchymal transition (EMT) between NCI-H520 and NCI-H520R was clearly evident, with mesenchymal markers (e. g. vimentin, N-cadherin) present in the parental line and epithelial markers (e. g. E-cadherin) in the resistant line (Supplementary Figure 1A and 1B). A number of antibodies specific for epigenetic marks were included in this proteomic analysis. Noteworthy was an approximately 13-fold elevation in Histone H4 - monomethyl Lys20 in NCI-H520R over NCI-H520 cells. HH4 methylation has been associated with enhanced DNA damage repair and protection against replication stress [27, 28]. Other interesting changes in protein levels included cyclin' A2 and HLA-ABC elevation in NCI-H520R versus NCI-H520 (Supplementary Figure 1A).

**Figure 7:**
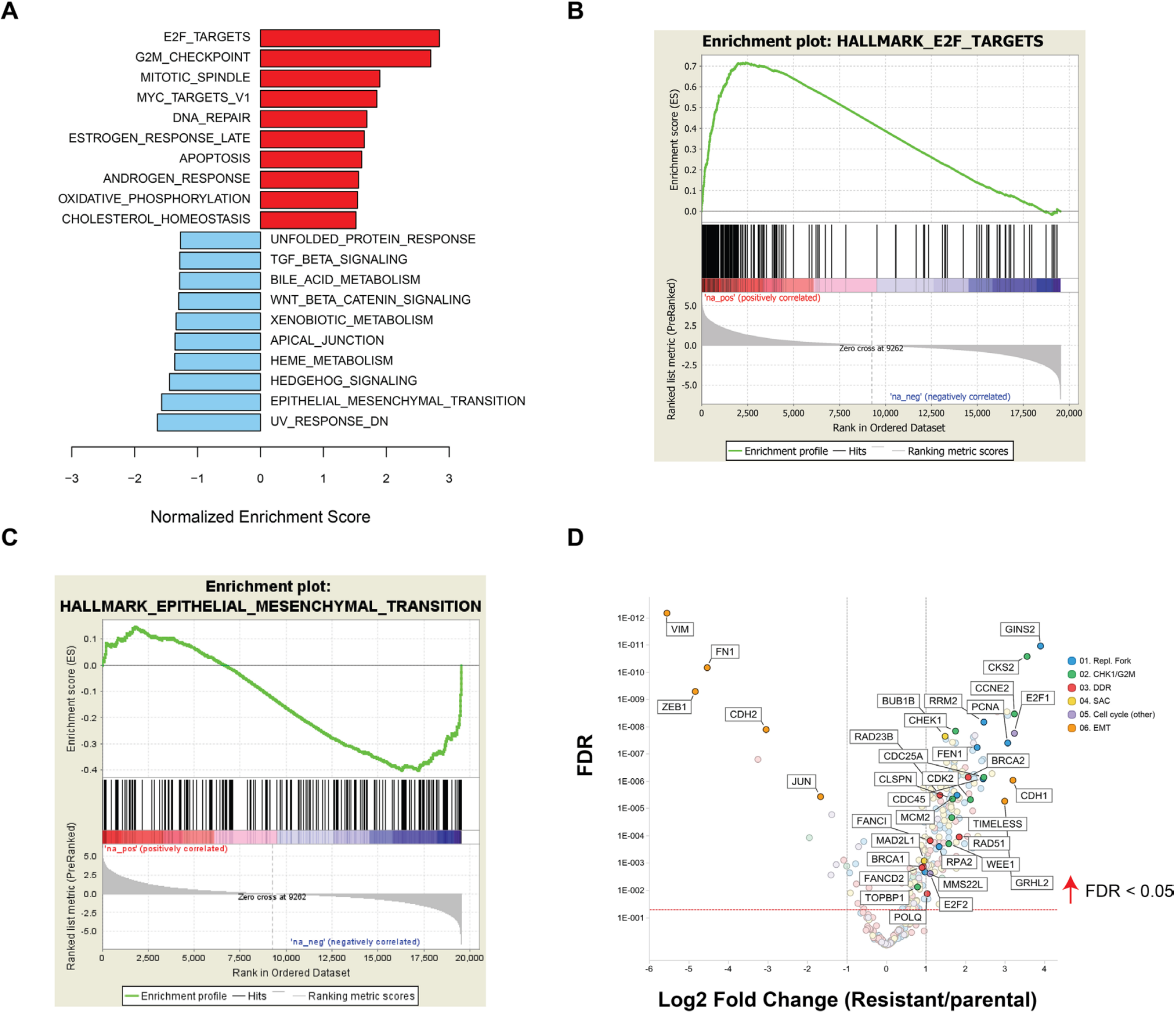
GSEA of differentially expressed genes between NCI-H520 and NCI-H520R cell lines. (**A**) Bar plot showing top upregulated (red) or downregulated (blue) gene sets in resistant versus parental tumor cell lines and representative enrichment plots highlighting key hallmarks associated with upregulated (**B**) or downregulated genes (**C**) in NCI-H520R over NCI-H520 tumor lines. (**D**) Volcano plot showing representative differentially expressed genes in NCI-H520 parental versus NCI-H520R prexasertib-resistant cells (FDR<0.05).

The drug resistance phenotype following chronic prexasertib treatment of NCI-H520R was investigated by next-generation sequencing (NGS). Exome analysis did not identify genetic variants that could readily account for the resistance mechanism and no mutations were identified in *CHEK1* nor in any other genes corresponding to CHK1-related pathways. In contrast, microarray analysis revealed a marked difference in transcriptional profile between NCI-H520R and NCI-H520 with 1,999 genes displaying >1.5-fold change and 2,052 genes displaying <-1.5-fold change in resistant versus parental (FDR<0.05) (Supplementary Table 7). GSEA revealed that the E2F/G2M/SAC signature was upregulated, with the top three statistically significant gene sets being E2F targets, G2M checkpoint, and mitotic spindle assembly (FDR< 2E-34) gene sets and MYC signaling and DDR noted as high-ranking pathways (Figure 7A, 7B). Inspection of some of the E2F/G2M/SAC genes displaying upregulated expression in resistant/parental H520 models revealed similarities to those observed tracking with prexasertib sensitivity in the pan-cancer cell line panel and the xenograft study (Figures 4A, 7B, Supplementary Tables 3, 6, 8). Examples of these include core replication fork genes (*MCMs*, *RPA2*), CHK1 pathway activation (*TIMELESS, CLSPN*, *CHEK1, CDC25A, WEE1, CKS2*) and DDR (*BRCA2, RAD51*). Genes down regulated in H520 resistant/parental were predominantly mesenchymal markers (*VIM, FN1, ZEB1*), consistent with the mesenchymal/epithelial shift revealed by proteomic analyses (Supplementary Figure 1).

### Transcriptional features corresponding to innate immunity appear in the acquired prexasertib-resistant phenotype

**Figure 8:**
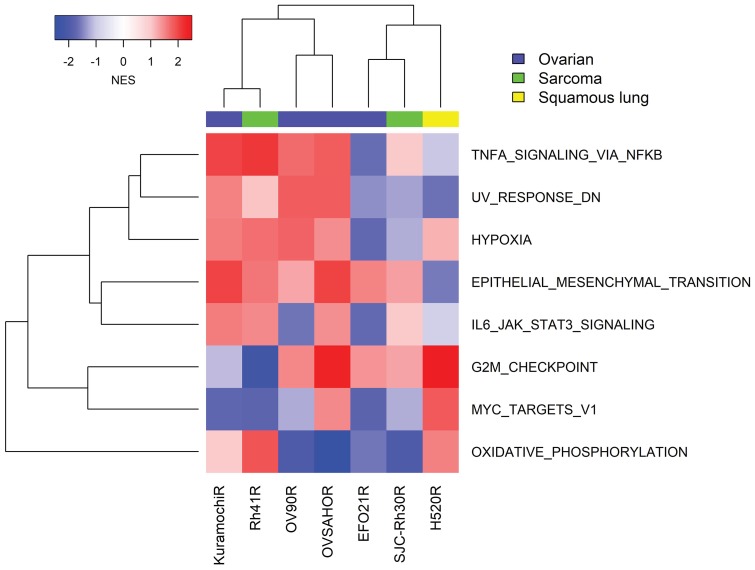
GSEA of transcriptomic data from seven cancer cell lines with acquired resistance to prexasertib. Gene expression analysis was carried out on replicate (mostly triplicate) parental/resistant cultures using RNASeq (four ovarian cancer cell lines) and microarrays (NCI-H520, SJC-Rh30 and Rh41). Expression data was normalized as described in Methods and subject to GSEA as described by Subramanian [19].

To understand whether other mechanisms of resistance existed beyond the E2F/G2M/SAC signal identified in NCI-H520R, we generated additional resistance models in tumors of interest to the clinical program including alveolar RMS (SJC-Rh30, Rh41) and ovarian cancer (Kuramochi, OVSAHO, OV90, EFO21) cell lines. All were established using an *in vitro* drug concentration escalation protocol except for SJC-Rh30 resistant clones which were isolated from a resistant tumor following *in vivo* prexasertib treatment. All models were investigated by GSEA of transcriptomic data. To identify generalizable features, a stringent filter was imposed to gene sets present in at least 5 out of 7 models at an FDR<0.05. G2M emerged as a signal of sensitivity in four additional cell lines besides NCI-H520R, while E2F and SAC could be observed as a significant gene sets when using the less stringent 4 out of 7 models filter. In contrast with NCI-H520R that displayed evidence of mesenchymal-epithelial transition (MET), other models of acquired resistance exhibited EMT markers. Interestingly, four of the resistant models displayed increased TNF alpha/NFκB and IL6-JAK-STAT3-associated genes when compared to their parental counterparts (Figure 8), suggesting that innate immunity is associated with the resistant phenotype.

**Figure 9:**
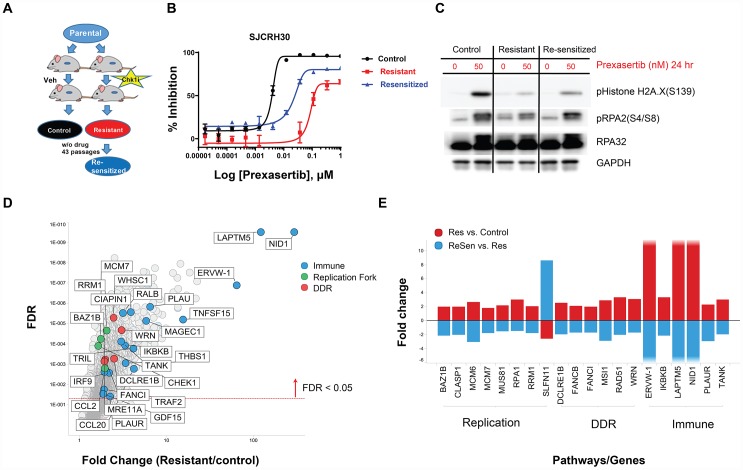
*In vivo* establishment and genomic characterization of acquired resistance to prexasertib in the aRMS SJC-Rh30 xenograft model. (**A**) Schematic of the method used to generate prexasertib-sensitive and –resistant cultures from SJC-Rh30 xenografts. (**B**) Viability of parental SJC-Rh30, SJC-Rh30R, and SJC-Rh30reS cells assayed following prexasertib treatment and corresponding levels of markers of DNA damage (γH2AX) and replication stress (pRPA2, S4/8) following drug treatment (**C**). (**D**) Change in RNA expression levels between SJC-Rh30R and SJC-Rh30 (red) and SJC-Rh30R and SJC-Rh30reS (blue). (**E**) Comparison of fold change in expression values for selected DDR, immune and replication fork genes between SJC-Rh30R and SJC-Rh30reS cells.

A previous study from our laboratory published by Lowery *et al*. [23] showed tumor regrowth upon prexasertib withdrawal in the pediatric aRMS CDX model SJC-Rh30, suggesting the development of acquired resistance to CHK1 inhibition or regrowth of residual tumor. To address the possibility that prexasertib treatment can induce acquired resistance *in vivo*, mice bearing SJC-Rh30 xenografts were treated with vehicle or with prexasertib using the study design shown in Figure 9A. Following the 28-day dosing interval, animals were maintained in a drug-free state and tumor regrowth was observed. Upon a second 28-day dosing interval, no evidence of tumor growth inhibition was observed suggestive of the development of acquired drug resistance. Cell lines were established from a tumor from a vehicle-treated animal (and presumably sensitive to prexasertib; SJC-Rh30) and a tumor which regrew following prexasertib treatment (SJC-Rh30R). The established tumor lines were sub-cultured in the presence of drug (50nM) and evaluated for drug response using a cell viability assay (CTG) identifying a marked difference in IC_50_ between tumor lines established control animal versus drug-treated animals (0.0047 μM versus 0.37 μM) (Figure 9B). In addition, SJC-Rh30R could be re-sensitized to prexasertib after subculture for 43 passages in the absence of drug (SJC-Rh30reS), though not to the same degree as the parental SJC-Rh30 cells (0.026nM) (Figure 9B). SJC-Rh30, SJC-Rh30R, and SJC-Rh30reS showed the expected increases in pRPA2 and γH2AX following prexasertib treatment, with SJC-Rh30reS appearing to have intermediate expression of these markers when compared to the parental or resistant lines (Figure 9B). Exome sequence analysis did not provide any evidence for mutations being a mechanism driving resistance, consistent with the observed reversibility of resistance. Similar to other prexasertib-resistant cell lines, transcriptomic analysis proved more informative than exome genetic analysis. Genes with functions in replication fork, DDR and innate immunity were upregulated in the drug-resistant state relative to the parental SJC-Rh30 model, a trend that was reversed in SJC-Rh30reS (Figure 9D and 9E; Supplementary Table 9).

**Figure 10:**
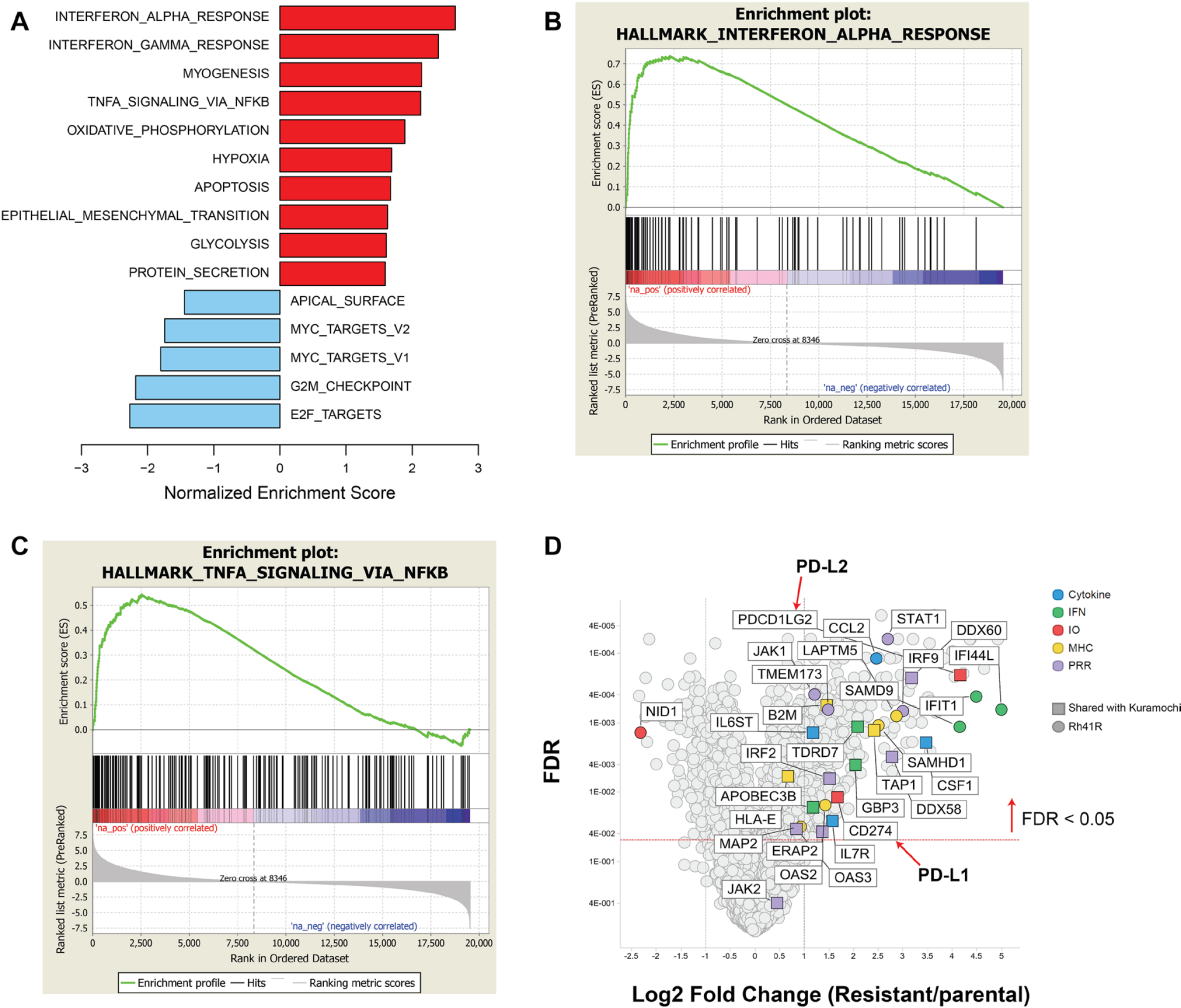
GSEA of differentially expressed genes in prexasertib-resistant aRMS Rh41R compared to parental Rh41 cells. (**A**) Upregulated (red bars) or downregulated (blue bars) gene sets in Rh41R versus Rh41. (**B** and **C**) GSEA plots for top-scoring immune-related gene sets associated with drug resistance. (**D**) Volcano plot showing representative innate immunity and other immune-related genes as fold change in Rh41R versus Rh41 (FDR<0.05).

Similar to SJC-Rh30, Rh41 shows exquisite sensitivity to prexasertib monotherapy both *in vitro* and *in vivo* [23]. Independent cultures of Rh41 were subjected to either DMSO (*n* = 5) or prexasertib (*n* = 5; Rh41R) over 6 weeks using concentration-escalation protocol. The individual cultures attained a drug-resistant state in near synchrony and displayed equivalent IC_50_ shifts and decreased pRPA2 and γH2AX (Supplementary Figure 2). Striking upregulation of multiple immune-related pathways was observed across all Rh41R cultures (Figures 8 and 10; Supplementary Table 10). Similar to previous findings in the pan-cancer cell line panel, multiple components of the innate immunity/STING pathway (including PRR, IFN signaling, MHC presentation, and PD-L1-mediated immune checkpoints) were identified as at least two-fold higher in Rh41R (FDR< 0.05) (Figure 10). Interestingly, GSEA of RNASeq data from resistant/parental pairs of ovarian cancer cell lines (Kuramochi, OVSAHO, OV90, and EFO21) generated using a similar concentration-escalation protocol also identified immune-related signals (Figure 8, Supplementary Table 11). EMT, the NFκB pathway, and IFN-alpha signaling were the top pathways upregulated in resistant versus parental tumor cell lines.

The recurrent appearance of innate immunity-related genes in the setting of acquired resistance to prexasertib prompted a closer examination of the transcriptomic data for evidence of common biological pathways (Supplementary Table 12). Three resistant cancer cell lines displayed upregulation of endogenous retroviruses, consistent with an interferon response unleashed by chronic prexasertib treatment. Multiple innate immunity-related genes were observed in models of both acquired resistance and innate resistance to prexasertib, suggesting that upfront upregulation of innate immunity may attenuate prexasertib response. An antibody recognizing a common epitope for HLA-AB C in the Digiwest proteomic array detected elevated expression levels in NCI-H520R compared to parental cells (Supplementary Figure 1) consistent with the transcriptomic data (Supplementary Table 12).

### Functional genomic studies to identify causal effects in genes corresponding to resistance to prexasertib

**Figure 11:**
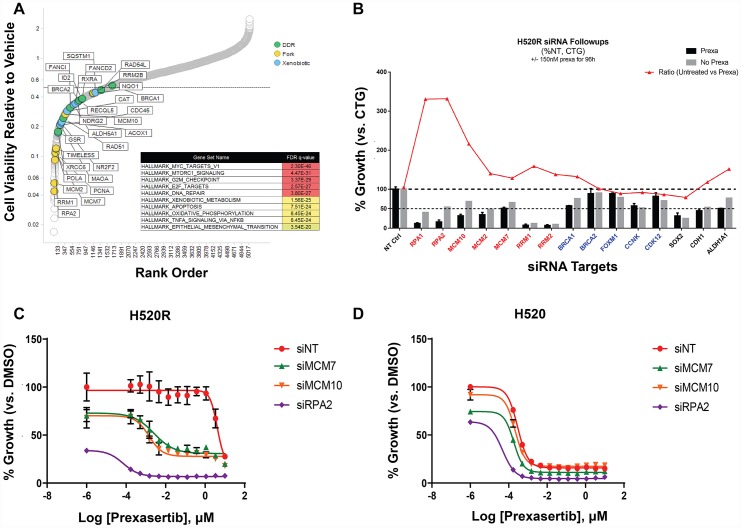
Short-hairpin (sh) modifier screen applied to NCI-H520R to identify genes capable of sensitizing cells to prexasertib treatment upon knockdown. (**A**) Rank order of genes with greater than 50% effect on cell viability of the resistant tumor line induced by on-target knockdown normalized to non-target control (calculated from the geometric mean of short-hairpins; Supplementary Table 13). (**B**) Tertiary follow-up screen centered on 15 hits identified in the primary screen; y-axis corresponds to the percent effect on cell viability (as measured by the CTG assay) of on-target/non-target control established for DMSO (grey bar) or prexasertib-treated cells (black bar); red tracing depicts the ratio of DMSO/drug treatment effect. Concentration-response (10-point curve, ranging from 1 nM-1 μM) of NCI-H520 (**C**) and NCI-H520R cell viability (**D**) as a function of siRNA exposure.

To uncover genes mechanistically linked to the prexasertib-resistant phenotype, a functional genomic screen was conducted to identify potential sensitizers in the NCI-H520R cell line. Resistant cells were infected with a shRNA library comprising 5,043 genes, selected with puromycin, and treated with prexasertib at the IC_20_ drug concentration. NGS was carried out on genomic library barcodes to identify genes conferring drug sensitization. A total of 1,531 genes induced a phenotypic effect >50% on drug sensitization (Figure 11A). GSEA revealed an enrichment for MYC targets, G2M checkpoint, and E2F targets as the most statistically significant genes sets which sensitized NCI-H520R cells to prexasertib upon knockdown (FDR< 7E-20) (Figure 11A). Inspection of these gene sets revealed a distinct enrichment for components of the replication fork and genes implicated in DDR, similar to that seen in our resistant cancer cell lines and xenograft models. A secondary screen was run on 40 hits (chosen based on magnitude of effect (>50% of NT control) and biological function including mechanism-proximal, e. g. replication fork, DDR and mechanism-distal, e. g. ALDHA1 followed by a tertiary screen on 15 hits (Figure 11B). The single-stranded DNA-binding protein encoding genes, *RPA1* and *RPA2* as well as several minichromosome maintenance complex genes (*MCM10*, *MCM2*, and *MCM7*) induced the strongest sensitization effect on NCI-H520R upon siRNA-mediated knockdown relative to the non-target control. The tertiary screen demonstrated that knockdown of *BRCA1, BRCA2, FOXM1*, *CCNK*, and *CDK12* did not sensitize NCI-H520R cells to prexasertib. The absence of sensitization to prexasertib with *BRCA1* knockdown in this experiment parallels observations made with TNBC tumor lines where depletion of this gene had no effect on prexasertib sensitivity (Figure 1).

**Figure 12:**
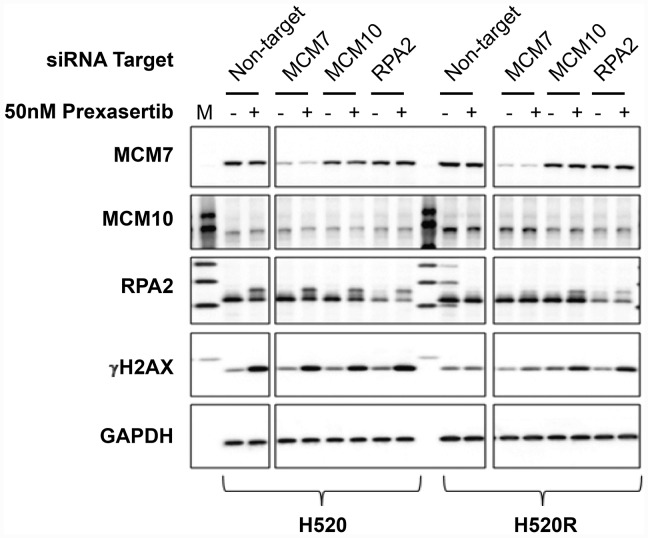
Effect of key replication fork gene knockdown on markers for replication stress and DNA damage measured in extracts derived from parental and prexasertib-resistant NCI-H520 tumor lines. pRPA2 (S2/S4) is visible a slower-migrating band on Western blot relative to its non-phosphorylated form. "M" refers to molecular weight marker on the first lane.

To further characterize NCI-H520 and NCI-H520R following siRNA-mediated knockdown of replication fork components, cells were transfected with non-target control or with siRNA targeting MCM7, MCM10, or RPA2. All three replication fork genes sensitized the resistant tumor line to prexasertib upon knockdown but had no effect on parental cell sensitivity (Figure 11C, 11D). We examined the levels of γH2AX and pRPA2 of non-target and on-target (*MCM7*, *MCM10,* and *RPA2*) siRNA transfections in NCI-H520 or NCI-H520R cells treated with DMSO or prexasertib. Increased γH2AX and pRPA2 signals were evident in cells treated with on-target relative to non-target siRNAs, supporting a mechanistic role for these replication fork genes in the prexasertib-resistant phenotype (Figure 12). Higher protein levels of MCM10 could be observed in NCI-H520R compared to the parental line (Figure 12), consistent with RNASeq data (Supplementary Table 7).

### Knockdown of cyclin A2 induces a profound desensitizing effect to prexasertib

**Figure 13:**
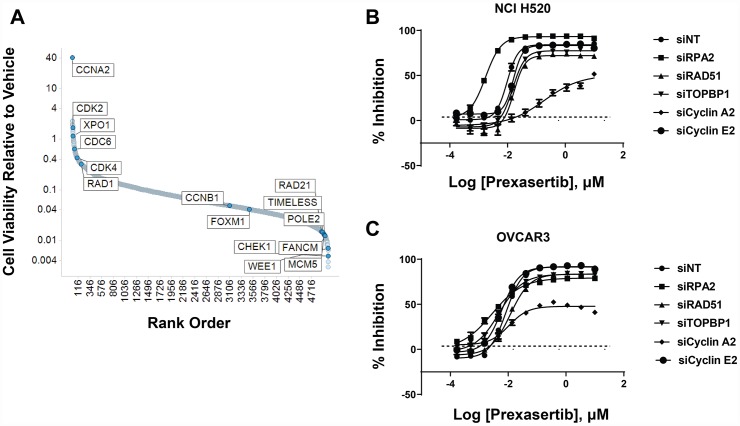
shRNA modifier screen applied to the NCI-H520 cell line identified genes that induced a sensitizing or de-sensitizing effect upon knockdown. (**A**) Y-axis corresponds to the ratio of the geometric means for prexasertib over DMSO. One corresponds to no effect, >1 shows hits that de-sensitize and <1 hits that sensitize to prexasertib upon knockdown. Hits from primary screen are shown in Supplementary Table 14. Confirmation of selected hits from shRNA screen using siRNA knockdown experiments in NCI-H520 (**B**) and the ovarian cancer cell line OVCAR3 (**C**).

NCI-H520 cells were subjected to shRNA-mediated knockdown using a short hairpin lentiviral library to identify genes that promote resistance to prexasertib upon downregulation. Following transduction with this library, puromycin-selected NCI-H520 cells were treated with DMSO or prexasertib at the IC_90_ drug concentration. NGS of the bar-coded library was carried out to identify genes conferring effects on cell viability. *CCNA2* emerged as an unexpected outlier in inducing a profound de-sensitizing effect upon knockdown (Figure 13A). siRNA-mediated *CCNA2* knockdown confirmed this observation by right shifting the IC_50_ of response to prexasertib in NCI-H520 cells (Figure 13B) as well as in the ovarian tumor line OVCAR3 (Figure 13C). Cyclin A2 has several non-cell cycle roles, including its function in stabilizing MRE11A by binding to its 3′UTR resulting in elevated protein levels [29] and MRE11A has been shown to be required for prexasertib-induced cytotoxicity [30]. However, *CCNA2* knockdown did not change MRE11A protein levels in neither NCI-H520 nor in OVCAR-3 tumor cells (Supplementary Figures 3 and 4).

To validate these findings, a different tumor line, high-grade serous ovarian OV90, was interrogated using CRISPR methodology to identify prexasertib de-sensitizers genes. The findings from the CRISPR screen were remarkably similar to those obtained in the de-sensitizing shRNA screen with NCI-H520 cells. Knockout of replication fork-associated genes enhanced the sensitivity of OV90 to prexasertib and knockout of DREAM complex genes, *CDC25A*, *CDK2*, *LIN54*, *CCNA2,* had the largest de-sensitizing effect (Supplementary Figure 5 and Supplementary Table 15).

## DISCUSSION

Approximately 10-15% of patients across separate clinical trials (NCT01115790, HNSCC, SCC; NCT02735980, SCLC; NCT02203513 HGSOC) have had some evidence of clinical benefit in response to prexasertib monotherapy. Prexasertib is a small molecule kinase inhibitor displaying high selectivity for CHK1 followed by CHK2 [5]. An initial search for predictive biomarkers in these patients revealed a signal centered on cyclin E1, such as LOF mutations in the E3 ubiquitin ligase *FBXW7* [12] and elevation of cyclin E1 protein levels [13]. Here, we confirmed that *FBXW7* knockdown is associated with elevation of cyclin E1 protein levels, CDK2 activation, CHK1 signaling, and increased RS (as measured by pRPA2) and DNA damage (evidenced by γH2AX). Relative to its non-target control, *FBXW7* knockdown enhanced the cytotoxic activity of prexasertib, supporting the hypothesis that *FBXW7* LOF mutations play a role in driving clinical response. These observations are in agreement with the known mechanism of action of prexasertib which involves hyper-activation of CDK2 arising from the stabilized CDC25A protein levels [5, 31]. Importantly, we observed one cancer cell line in which elevated cyclin E1 caused by knockdown of *FBXW7* did not appear to activate CDK2 (as measured by pNPM). Signaling context, therefore, may impact the predictive power of cyclin E1 as a biomarker of response to prexasertib. Hauge *et al.* [32] reported that excessive p21 limits S phase DNA damage caused by the WEE1 inhibitor MK1775 (adavosertib), again supporting the notion that negative regulators of CDK2 may limit cytotoxicity to agents that target the ATR-CHK1-WEE1 pathway. Although these observations have supported cyclin E*-*centered hypothesis for prexasertib response, clinical actionability of this marker is limited by insufficient predictive power [12, 13]. In addition, our preclinical data further supports this limitation in applicability as a single marker for patient selection.

To widen the search for mechanisms associated with prexasertib response, a preclinical, tumor type-agnostic search of such molecular signals was undertaken by associating drug response with molecular (DNA, RNA, protein) expression data across a large tumor cell line panel and a cohort of patient-derived xenografts. Using a statistical cutoff (FDR<0.05), no recurrent exome signals associated with response in the pan-cancer tumor panel nor across the xenograft models studied were observed. In contrast, RNASeq identified a statistically significant signal associated with prexasertib sensitivity that showed striking similarity between a pan-cancer cell line panel and a sarcoma/neuroblastoma *in vivo* xenograft model cohort. The top-ranking gene sets in the transcriptional sensitivity signature include E2F transcription targets, G2M, and spindle-associated checkpoint (SAC) genes (E2F/G2M/SAC signature). E2F and MYC family members play a crucial role not only as mechanisms driving tumor cell proliferation, but also as inducers of RS [33]. Paradoxically, these same transcription factors mitigate DNA damage by targeting the promoters of genes that play a key role in DDR and fork restart; Bertoli *et al.* [34] showed that E2F target genes are induced by RS and play a role in survival to RS. Similarly, MYC displays tumor-promoting (stimulation S-phase entry) and tumor-suppressing (DNA damage protection) mechanisms [33]. While E2F activity is required to drive cell pro-proliferative and RS-inducing signaling, paradoxically, E2F-dependent transcription plays a role in limiting DNA damage by stimulating transcription of genes associated with fork stability and DNA repair [34]. The pan-cancer E2F signature associated with prexasertib response, therefore, may represent a survival mechanism employed by tumor cells exhibiting high levels of OIRS.

We explored a complementary approach to uncover mechanisms driving response to prexasertib, namely the establishment and molecular and phenotypic characterization of a model with acquired resistance to prexasertib. We chose NCI-H520 based on prior clinical efficacy observed in squamous cancers [12]. Exome analysis failed to identify genetic variants that could provide clues as to how resistance to prexasertib arises. Microarray analysis, in contrast, identified the E2F/G2M/SAC signature previously observed in the pan-cancer panel and sarcoma/neuroblastoma xenograft study as a driver of sensitivity to prexasertib. This finding, at first glance, appears paradoxical as a signature associated with sensitivity to prexasertib would be unlikely to reappear as one associated with acquired resistance. The simplest explanation is centered on the concept of how tumor cells titrate mechanisms and build up buffering capacity to survive RS. The activation of an E2F transcriptional program provides a mechanism to survive RS [34]. The source of RS could be oncogene-driven mechanisms like increased or dysregulated cyclin E but they could also be drug-induced such as chronic prexasertib exposure, a mechanism known to trigger DNA damage [5, 18, 23]. A potential drug escape mechanism for tumor cells exposed to prexasertib would be to upregulate E2F/G2M/SAC gene expression providing a transcriptional context that would make them more tolerant to chronic RS. Consistent with our observations are recent findings by Bianco *et al*. [35] that overexpression of Claspin and Timeless, themselves E2F transcription factor targets, play a key role in ATR-CHK1 signaling pathway-mediated protection against RS in cancer cells.

In an effort to explore how common would an E2F/G2M/SAC signature be in the context of acquired resistance to prexasertib, we established additional resistance models. GSEA of transcriptomic data identified the same signature across additional tumor models (e. g. ovarian OVSAHO, EFO21, OV90; aRMS SJC-Rh30), while unexpectedly revealing a gene signature associated with innate immunity in others (e. g. ovarian Kuramochi; aRMS Rh41). A framework to reconcile these divergent findings takes into account two well-established facts: the primary MOA of prexasertib is centered around DNA damage (measured by γH2AX) [5], and DNA damage is associated with activation of innate immunity via the STING pathway [36]. A second important observation was identifying the innate immunity transcriptomic signature associated with primary resistance to prexasertib across the pan-cancer tumor cell line panel and in the sarcoma/neuroblastoma xenograft cohort. An intriguing clinical implication of this finding is that an inflamed or “hot” tumor may not respond as well to prexasertib as an immunologically “cold” tumor.

An increasing number of observations support a connection between DNA damage and immune response in the context of cancer. Treatment of small-cell lung cancer tumor lines with prexasertib (or with the PARP inhibitor olaparib) triggered expression of chemokines [37]. Similarly, PARP inhibition triggered STING-dependent anti-tumor immunity in *BRCA1*-deficient ovarian cancer tumor models [38] and in models of triple-negative breast cancer [39]. In addition, cis-platinum treatment in preclinical ovarian cancer models led to upregulated expression of MHC, several cytokines and chemokines, and other immune-related molecules [40]. Radiotherapy was found to be associated with upregulation of innate immunity [41]. *BRCA2* deficiency activated a cGAS/TNFα inflammatory signal [42]. The latter finding bears relevance to the prexasertib monotherapy trial in HGSOC patients where the efficacy readout gave the confounding result that some *BRCA*-mutant patients received less clinical benefit than *BRCA*^wt^ patients [13]. Accordingly, a STING-high tumor (as a consequence of BRCAness) would be expected to be less sensitive to prexasertib.

An important caveat to the aforementioned observations is that they represent associations of drug response and molecular signals but lack evidence to mechanistically link these markers to CHK1 biology. An shRNA functional genomic screen in NCI-H520R cell line identified a clear enrichment for replication fork genes as sensitizers of prexasertib, further confirmed by siRNA-mediated knockdown experiments. The presence of a remarkably similar E2F/G2M/SAC signature tracking with sensitivity in the pan-cancer cell line panel as well as in the xenograft cohort is consistent with this signature being representative of an adaptive mechanism to RS, whether this represents OIRS- or prexasertib-induced RS. Knockdown of ssDNA-protecting *RPA* genes and core components of the MCM helicase such as *MCM2*, *MCM7*, and *MCM10,* emerged as having the highest sensitization effects to prexasertib. In line with this observation, RPA and MCM proteins have been shown to act as buffers against RS in multiple studies [43–45].

Unexpectedly, in some of the prexasertib-resistant models the dominant signature was not the E2F/G2M/SAC signature but rather an innate immunity signal. While CHK1 mechanism-proximal genes such as *RPA* and *MCM* family members may have a role as modifiers of response to prexasertib as supported by our experimental evidence, it is far less obvious how immunity genes could have similar effects on prexasertib response. Closer inspection of the genes emerging from our shRNA modifier screen on the NCI-H520R model provide some clues suggesting a role for immune signaling pathway linking prexasertib sensitivity to DDR (Supplementary Table 13): *FOS* and *JUN*, which target expression of DDR genes [42]. *TRADD,* known to upregulate non-homologous end-joining DDR [46]; the urokinase receptor *PLAUR*, which activates CHK1 and RAD51 in response to DNA damage [47]; and multiple cytokines and their associated receptors that regulate DDR through STAT signaling [36]. In the clinical setting, a second mechanism that could potentially limit drug efficacy relates to the presence of immune checkpoints (e. g. PD-L1, PD-L2, others) that were induced by chronic prexasertib exposure. Further investigation for a role of innate immunity as a modifier of response to prexasertib is warranted.

Interestingly, siRNA-mediated knockdown of *CCNA2* had a profound de-sensitizing effect to prexasertib treatment. Cells with reduced *CCNA2* were found to have concomitant attenuation in both RS and DNA damage signals following exposure to prexasertib. A similar observation emerged from a CRISPR screen in another tumor cell line. *CCNA2* was also observed associated with sensitivity to prexasertib in the pan-cancer tumor cell line panel. The identification of *CCNA2* in two independent genomic screens across two different cell lines point to an essential role for this cyclin in prexasertib’s MOA. The simplest interpretation relates to the role of cyclin A2-CDK1/2 in replication origin firing in S phase [33].

The intricate relationship between RS, DNA damage, and immunity [36, 48–51] put the genomic signals observed in our study into context in preclinical models; however, it is not known whether they translate to the observed clinical efficacy or lack thereof of prexasertib in patients. The response rate for prexasertib monotherapy studies across three trials (NCT01115790, NCT02203513, NCT02735980) is approximately 10-15%, contrasting sharply with the broad spectrum of activity observed across preclinical models *in vitro* as well *as in vivo*. Our study suggests that tumors may utilize different pathways to overcome prexasertib-induced cytotoxicity. One pathway implicates replication fork components –upregulation of E2F transcription factor target genes such as *MCM* and *RPA* family members, among others. A second mechanism may involve activation of the STING/IFN response pathway, implicated in both primary and acquired resistance evident in our data. The STING/IFN signal detected in intrinsic resistance raises the possibility that prior genotoxic therapy, *BRCAness*, or other activating mechanisms prior to prexasertib may contribute to lower clinical efficacy. A STING/IFN-mediated prexasertib resistance mechanism could, in theory, be overcome by co-targeting STING or downstream effector molecules. Our findings are relevant to other targetable components of the CHK1 pathway (e. g. ATM, ATR, WEE1) as inhibitors of these protein kinases may, like prexasertib, stimulate a STING-mediated IFN response upon DNA damage. Although we cannot rule out a potential role of CHK2 in the observed biology, the enhanced selectivity of prexasertib on CHK1 over CHK2 [5] and the fact that DNA damage appears to arise as a consequence of CHK1 rather than CHK2 inhibition [5, 23], points to an ATR-CHK1 rather than ATM-CHK2 pathway mechanism.

## MATERIALS AND METHODS

### Cell lines and tissue culture

Pan-cancer tumor cell line panel (tumor lines = 572, 29 histologies) has been previously described [17]. Tumor cell lines used for drug resistance studies are shown in Supplementary Table 16. Cell viability assay was performed using CellTiter-Glow (Promega). Drug studies were conducted with LY2940930, the mesylate monohydrate salt of LY2606368 (prexasertib, Eli Lilly and Company) [5], and is referred to as prexasertib in this study.

### Resistant tumor cell line derivation

NCI-H520 (squamous lung) was subject to a drug concentration escalation protocol starting with 2 nM to final concentration of 75 nM over 60 days. A similar approach was utilized for resistant tumor line derivation for the other tumor lines investigated, time to resistance being variable across the models tested. Parental NCI-H520 and its prexasertib-resistant version are referred to in abbreviated form as H520 and H520R in figure titles.

### Establishment of resistance to prexasertib *in vivo*

The alveolar rhabdomyosarcoma tumor line SJC-Rh30 was implanted into an athymic mouse and tumor was allowed to grow. At day 23 post-implantation prexasertib dosing was initiated (prexasertib, 10mg/kg, (BIDx3 rest 4 days) x4). Dosing was stopped and tumor regrowth was observed. At day 62, dosing was started once again. (prexasertib, 10mg/kg, (BIDx3 rest 4 days) x4). No growth inhibition was observed on the second round of dosing. At day 91, tumors (from vehicle and drug treatment arms) were harvested and placed in culture media (50% FBS, 40% RPMI - ATCC 30-2001, and 10× antibiotic/antimycotic solution - Hyclone SV30079.01) for approximately 1 hour prior to processing. Tumors were transferred to 100mm dishes, washed with PBS and minced with a sterile scapel following addition of trypsin. Cells and tumor pieces were collected by centrifugation at 1,000 rpm for 7 min. Cells and tumor pieces were placed in a T75 flask in culture media (50% FBS, 40% RPMI – ATCC 30-2001, and 10x antibiotic/antimycotic solution - Hyclone SV30079.01), incubated overnight followed by change to fresh culture media (without removing the tumor pieces). Repeated the overnight incubation and changed media to 10% FBS containing 1x antibiotic/antimycotic solution. Cells grew following these manipulations. Sub-culturing involved a 1:2 split in the presence of 10 nM prexasertib.

### Proteomics

Western blot antibodies appear on Supplementary Table 17. Digiwest analysis [26] was carried using lysates from triplicate cultures. Antibodies used for Digiwest proteomics appear on Supplementary Table 8, column A.

### shRNA genomic screen

NCI-H520 prexasertib resistant cells (NCI-H520R) were infected with Cellecta Module 1 DecipherTM library targeting the signaling pathways (Cat #DHPAC-M1-P) at a multiplicity of infection of <1. The lentiviral based library is comprised of 5,043 genes with 5 to 6 plasmid pools per gene. Lentiviral particles were generated as described in the Cellecta manual. Infected cells were selected with puromycin for 72h. Following selection, cells were pooled, plated and treated with DMSO or prexasertib at an IC_20_ of 150nM, refreshing media during the experiment. Genomic DNA was extracted using the Qiagen kit (Cat # 13362) as described in the manual. The barcodes tagged to each shRNA were amplified by PCR and sequenced on Illumina NextSeq 500 according to Cellecta’s manual. The level of quality control for the screen was measured at the sequencing, sample and gene level. To ensure quality of sequencing, cluster density was performed to determine the distribution of sequencing points along the flow cells and number of reads. Higher cluster density defines higher reads and low error rates. Additionally, error rates of base calls were calculated using (1) PhiX control where 5 to 10% of PhiX library was spiked-in to introduce diversity. This enable discrimination of clusters that allows signal thresholds of base calls (2) Q30 values that measures the relationship of sequencing quality score and base call accuracy. The library used in this study has a Q30>90% which indicates a 99.9% base call accuracy. To measure the sample quality, correlation coefficient values (r) of all 3 replicates were calculated with values of >0.5. At the gene level, individual negative controls were assessed, and the ratio of these genes did not show any signal changes between treated and untreated control. Experiments were run in triplicates. Geometric mean of signal intensity was calculated for all 3 replicates. The shRNA effects values for each gene were the ratio of geometric mean signal intensity values to that of the signal intensity obtained from plasmids.

### CRISPR genomic screen

OV90 cells were transduced with pRCCH-CMV-Cas9-2A-Hygro lentivirus from Cellecta to generate stable and functional Cas9-expressing cells (OV90-Cas9). These OV90-Cas9 cells were then transduced with Cellecta’s 80K Human genome-wide CRISPR knockout library at a MOI of 0.5. Puromycin was added to transduced cells 48h later. Upon 72h of puromycin selection, two aliquots of 10^8^ cells were harvested as baseline control samples, while the remaining cells were divided into three groups, with duplicate samples each, or treated with DMSO, prexasertib at IC_20_ concentration, and prexasertib at IC_90_ concentration. All groups were cultured for an additional 17 days (e. g. 10 doubling times of OV90 cell line), during which process cell culture was propagated and culture medium refreshed every 3-4 days. At the end of the screen, genomic DNA was extracted from 10^8^ cells of each biological replicate using the Qiagen kit (Cat # 13362). Single-guide RNAs were amplified by PCR and sequenced on Illumina HiSeq 2500.

Sequencing reads from the fastq files were processed to the guide RNA count table using an in-house pipeline (available upon request). In brief, sequencing adapters were removed by cutadapt [52] (version 1.18, with the following parameters: -n 10 -O 10 -e 0.1–match-read-wildcards) and reads with exactly 20 bases were retained for further analysis. After mapping with BWA [53] (version 0.7.17, with the following parameters: -T 15 -k 10 -L 100 -O 100) to the guide RNA reference, reads aligned with no mismatch were tallied for each guide. The count tables from all crispr libraries were merged and further analyzed by MAGeCK [54] computational tool, specifying the negative control sgRNAs for normalization and generation of the null distribution for MAGeCK with the “norm-method control” option, with gene copy number correction. The cell line gene copy number information was obtained from COSMIC [55]. Tabulated data (Supplementary Table 15 shows the calculated values for four conditions: Baseline (hereafter short for Base), DMSO, IC_20_ and IC_90_. Therefore five comparisons using MAGeCK were derived: DMSO_vs_Base, IC_20__vs_Base, IC_90__vs_Base, IC_20__vs_DMSO, IC_90__vs_DMSO. For each comparison, we report two statistics on gene level: the log2-fold-change (hence the prefix “log^2^FC”) and the signed log^10^ transformed RRA score (hence the prefix “log^10^Score”).

### RNA sequencing and data processing

Microarray: total RNA was isolated from replicate (mostly triplicate) cultures using Zymo Research kit (cat # R2051). Transcriptome analysis was performed using Clariom-S HTA (Thermofisher p/n 902927). RNASeq: total RNA was isolated and processed at WuXi NextCODE for RNA deep sequencing. The cDNA library was generated using TruSeq Stranded mRNA Library Prep Kit (Illumina, San Diego, CA, USA) and sequenced on Illumina HiSeq4000 systems with 150-bp paired-end configuration according to the manufacturer’s protocol. RNASeq data were subjected to an alignment and QC pipeline developed at Eli Lilly and Company. Briefly, pre-processed FASTQ files were aligned to GRCh37/hg19 human reference genome using the GSNP alignment algorithm. Then base quality/base composition, heterologous organism contamination, adapter content, mapping rate/mapped read counts, 3′ bias, template length, and rRNA/mitochondrial content were checked. All the samples passed RNASeq QC assessment. The aligned data were subjected to a “rollup” pipeline developed at Eli Lilly and Company for gene expression measurement. First, exon reads of multiple assays from the same libraries were aggregated. Then exons with less than 10 reads in more than 80% of samples were excluded. The gene expression was determined using a robust linear model based on all exon and junction reads. Last, quantile normalization was performed for each sample. Raw. CEL files were subject to quantile normalization and FDR, fold change calculated using published methods [56]. Gene Set Enrichment Analysis (GSEA) was run against the Cancer Hallmarks as described by Subramanian *et al*. [19].

### Gene set enrichment analysis (GSEA)

GSEAPreranked tool (http://software.broadinstitute.org/gsea/index.jsp) was used to detect the enrichment of the hallmark gene sets in Molecular Signatures Database (MSigDB, http://software.broadinstitute.org/gsea/msigdb/index.jsp) by a given dataset [19]. In the prexasertib acquired-resistant cell line models, all the genes were ranked according to P value and fold change (from up to down-regulation) derived from differential expression analysis before inputting into GSEA. Therefore, the positive enrichment score indicates an enrichment of the hallmark set by up-regulated genes in acquired resistant line, while the negative enrichment score indicating an enrichment by down-regulated genes. In the pan-cancer tumor cell panel and sarcoma PDX model, all the genes were ranked according to association with prexasertib sensitivity derived from correlation analysis of gene expression and prexasertib IC_50_, ordering from genes associated with high resistance to those associated with high sensitivity. Therefore, the positive enrichment score indicates an enrichment of the hallmark set by prexasertib-resistant genes, while the negative enrichment score indicates an enrichment by prexasertib-sensitive genes. In prexasertib acquired-resistant cell line models, 31 hallmark sets were selected with FDR<0.25 in at least five of the cell line models. The normalized enrichment scores were used for unsupervised hierarchical clustering and heatmap generation in R using the gplots package. In the pan-cancer tumor cell panel and sarcoma PDX/CDX model, 19 hallmark sets were selected with FDR<0.05 in both datasets. Clustering and heatmap were generated as described above.

### Genomic association with drug response

Prexasertib drug response across the pan-cancer tumor cell line panel was carried as described by Gong *et al.* [17] using the tumor lines listed on Supplementary Table 1. Efficacy studies were conducted as described by Lowery *et al.* [23] using the models listed on Supplementary Table 4. Gene expression/drug response association studies were based on RNAseq data generated across the pan-cancer tumor cell line panel or accessed from the xenograft model providers (START (https://www.startthecure.com/) and Champions Oncology (https://championsoncology.com/)). The raw gene expression data was normalized as described in the "RNA sequencing and data processing" section of Material and Methods. P values were determined by a linear regression model to test the significance of the association between prexasertib response and gene expression. Prexasertib IC50 was modelled on a log scale and, where applicable, a generalized Tobit model was applied to account for censored IC50 data [57]. False discovery rate (FDR) was then computed using Benjamini Hochberg method [56]. Data analysis was carried out in statistical soft-ware R (https://www.R-project.org/).

#### Geo accession numbers

GEO numbers for microarray and RNAseq data are as follows: Microarray data: GSE143007; RNAseq data: GSE143152.

## SUPPLEMENTARY MATERIALS

































## Abbreviations

RS: Replication Stress
OIRS: Oncogene-Induced Replication Stress
DDR: DNA Damage Repair
EMT: Epithelial Mesenchymal Transition
MET: Mesenchymal Epithelial Transition
PRR: Pattern Recognition Receptors
STING: Stimulator of Interferon Genes
IFN: Interferon
CDX: Cell Line-Derived Xenograft
PDX: Patient-Derived Xenograft
TNBC: Triple-Negative Breast Cancer
HGSOC: High-Grade Serous Ovarian Cancer
NGS: Next-Generation Sequencing
GSEA: Gene Set Enrichment Analysis
shRNA: Short Hairpin RNA
siRNA: Small Interfering RNA
MOA: Mechanism of Action
