# Supplementary material for: A pan-cancer transcriptome analysis identifies replication fork and innate immunity genes as modifiers of response to the CHK1 inhibitor prexasertib
Source: Oncotarget. 2020 Jan 21;11(3):216–36. doi: 10.18632/oncotarget.27400 (PMC6980627; doi:10.18632/oncotarget.27400)
Supplement: Supplementary file 1 [file oncotarget-11-216-s001.pdf]

A pan-cancer transcriptome analysis identifies replication fork and innate immunity genes as modifiers of response to the CHK1 inhibitor prexasertib

SUPPLEMENTARY MATERIALS

REFERENCE

1. Gong X, Litchfield LM, Webster Y, Chio LC, Wong SS, Stewart TR, Dowless M, Dempsey J, Zeng Y, Torres R, Boehnke K, Mur C, Marugan C, et al. Genomic Aberrations that Activate D-type Cyclins Are Associated with Enhanced Sensitivity to the CDK4 and CDK6 Inhibitor Abemaciclib. Cancer Cell. 2017; 32:761–76.e6. https://doi.org/10.1016/j.ccell.2017.11.006. [PubMed]

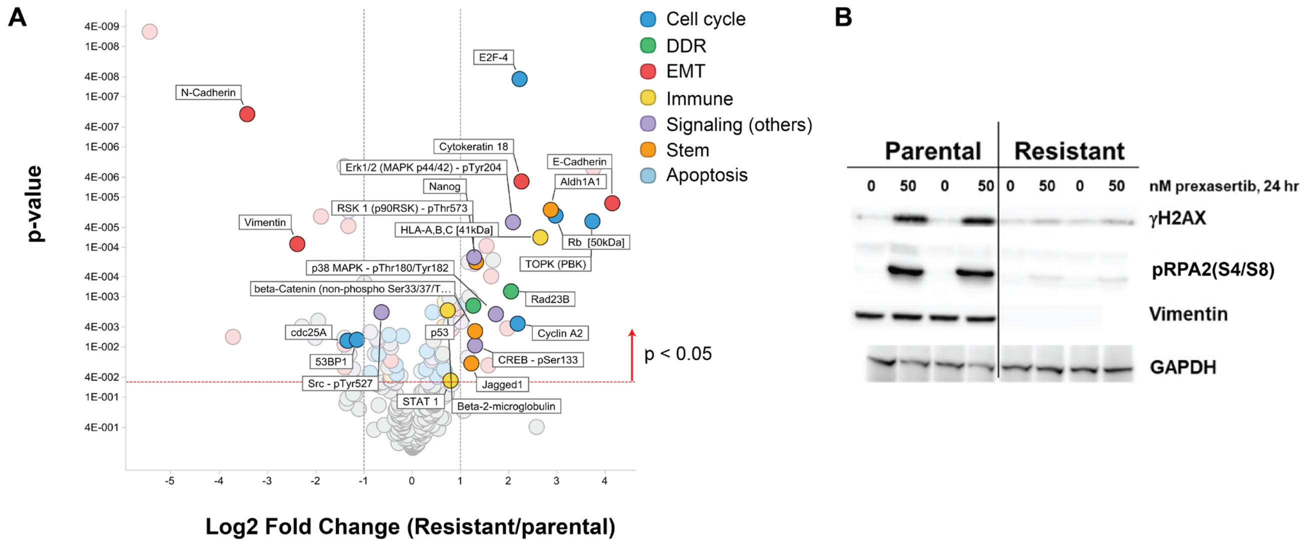

Supplementary Figure 1: Proteomic profile of parental and prexasertib-resistant NCI-H520 tumor lines. Protein levels measured from cellular extracts using Digiwest technology (**A**) or Western blots (**B**).

A

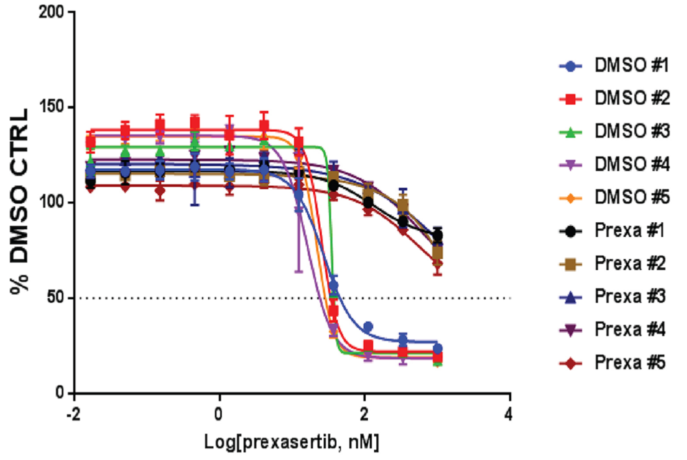

	DMSO #1	DMSO #2	DMSO #3	DMSO #4	DMSO #5	Prexa #1	Prexa #2	Prexa #3	Prexa #4	Prexa #5
IC50	53.54	29.35	36.42	21.38	25.82	4142	1873	2882	1808	1896

B

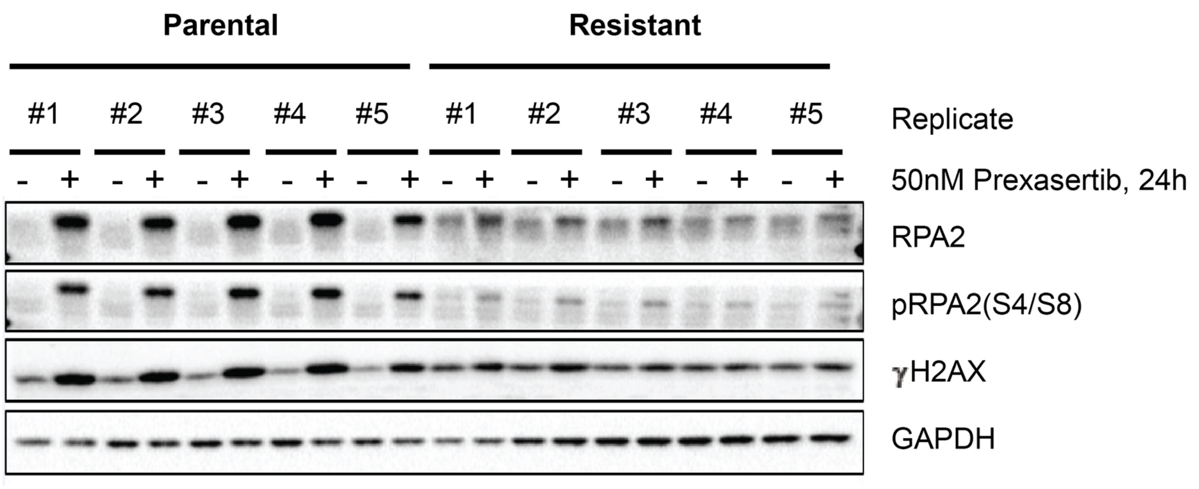

Supplementary Figure 2: Phenotypic characterization of prexasertib resistance in the aRMS tumor line Rh41. (**A**) IC_{50} determination using a cell viability assay (Cell Titer Glow) in 5 independent cultures of Rh41 and their corresponding prexasertib-resistant models with corresponding IC_{50} values. (**B**) Levels of replication stress (pRPA2) and DNA damage (γH2AX) in control (DMSO) or drug-treated parental or drug-resistant cultures.

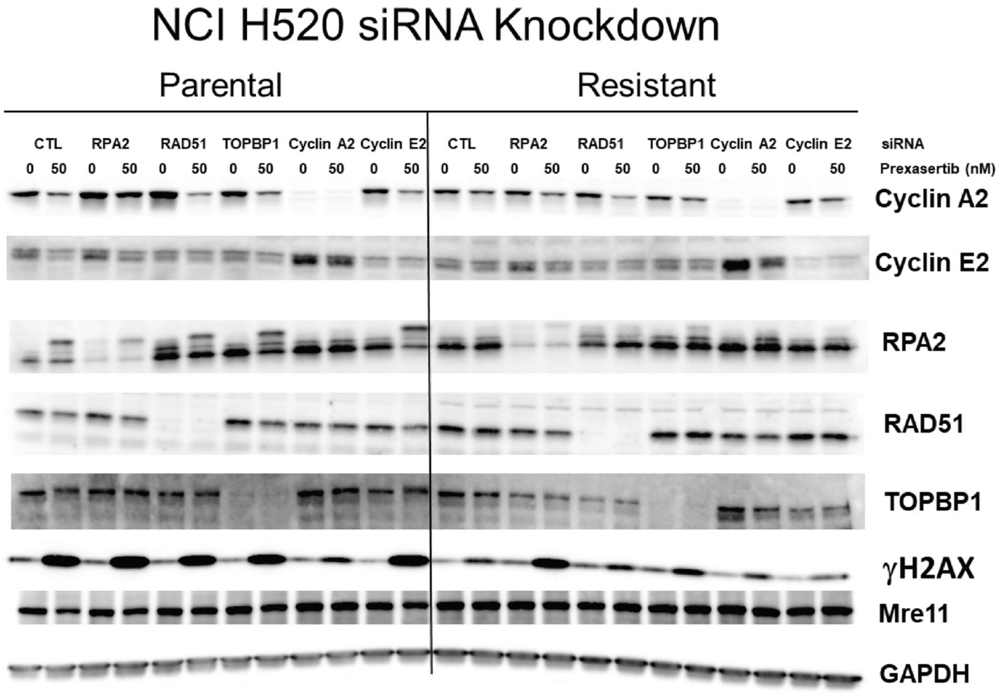

Supplementary Figure 3: Proteomic analysis of siRNA-treated NCI-H520 tumor line. Effect on the levels of a marker of DNA damage (γH2AX) and Mre11A as a consequence of non-target (CTL) control or on-target (*RPA2, RAD51, TOPBP1, CCNA2, CCNE2*) established by Western blot in siRNA-treated NCI-H520 tumor line.

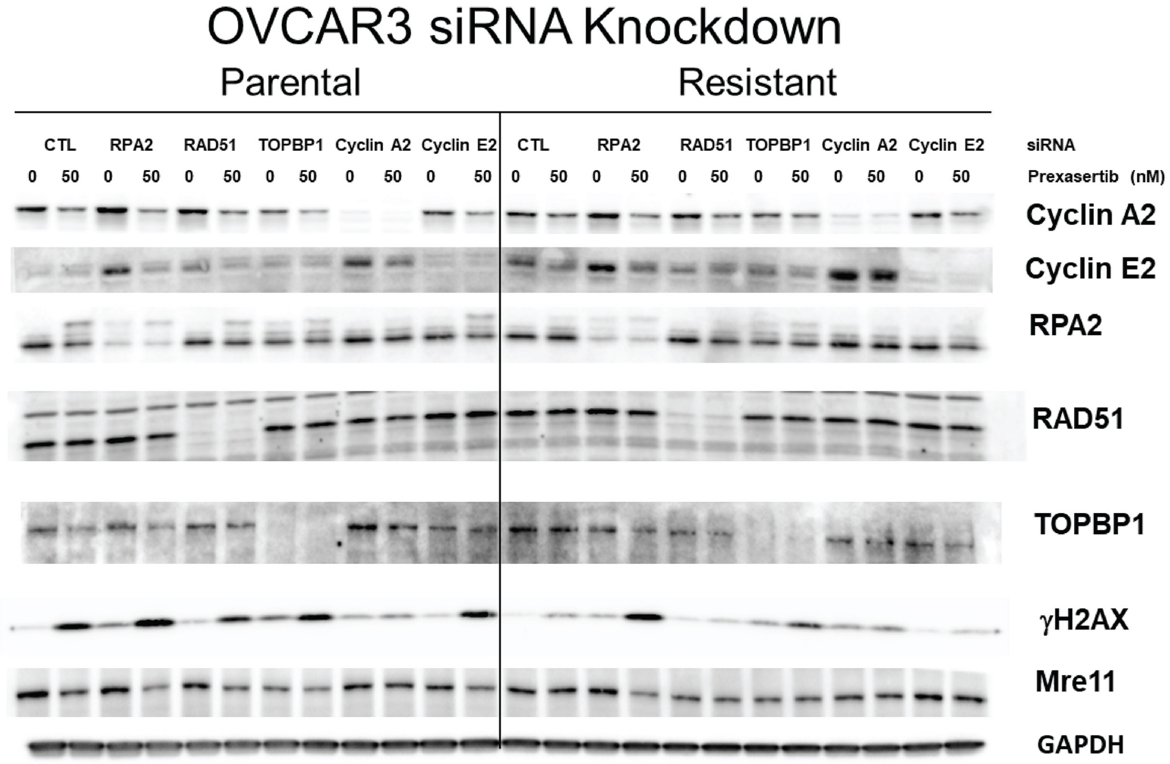

Supplementary Figure 4: Proteomic analysis of siRNA-treated OVCAR-3 tumor line. Effect on the levels of a marker of DNA damage (γH2AX) and Mre11A as a consequence of non-target (CTL) control or on-target (*RPA2, RAD51, TOPBP1, CCNA2, CCNE2*) established by Western blot in siRNA-treated OVCAR-3 tumor line.

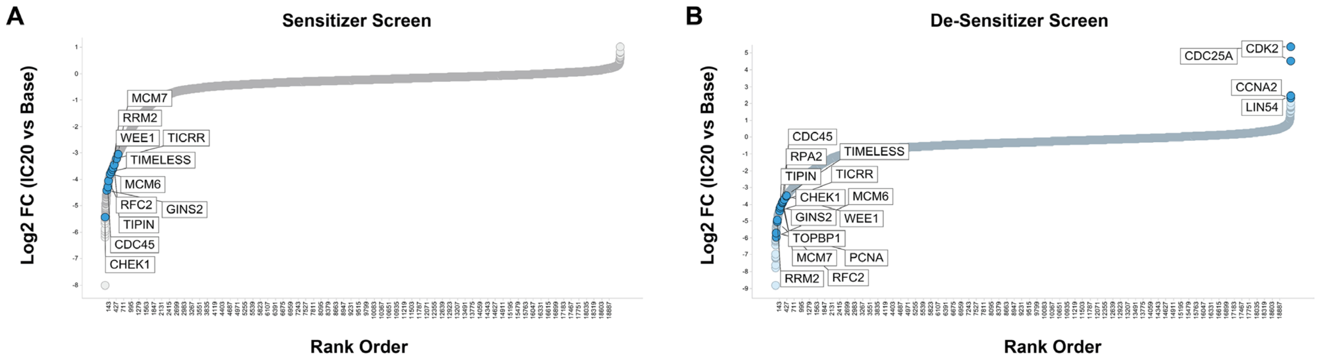

Supplementary Figure 5: CRISPR screen on the ovarian tumor line OV90. Genes having a sensitizing (**A**) or de-sensitizing effect (**B**) on prexasertib sensitivity upon knockout.

Supplementary Table 1: Activity of prexasertib across a pan-cancer tumor cell line panel. See Supplementary Table 1

Supplementary Table 2: Association between gene expression and prexasertib sensitivity across pan-cancer tumor cell line panel. See Supplementary Table 2

Supplementary Table 3: Association between gene expression and prexasertib resistance across pan-cancer tumor cell line panel. See Supplementary Table 3

Supplementary Table 4: Activity of prexasertib in pediatric and adult sarcoma/neuroblastoma PDX cohort and molecular features. See Supplementary Table 4

Supplementary Table 5: Association between gene expression and prexasertib sensitivity in sarcoma/neuroblastoma PDX cohort. See Supplementary Table 5

Supplementary Table 6: Association between gene expression and prexasertib resistance in sarcoma/neuroblastoma PDX cohort. See Supplementary Table 6

Supplementary Table 7: Differentially expressed genes between parental and prexasertib-resistant NCI-H520 tumor cell lines. See Supplementary Table 7

Supplementary Table 8: Proteomic profiling of parental and prexasertib-resistant NCI-H520 tumor cell lines. See Supplementary Table 8

Supplementary Table 9: Differentially expressed genes between parental and prexasertib-resistant Rh41 tumor cell lines. See Supplementary Table 9

Supplementary Table 10: Differentially expressed genes between resistant (Res) and parental (Par) and between re-sensitized (Re-sen) and resistant (Res) SJC-Rh30 tumor cell lines. See Supplementary Table 10

Supplementary Table 11: Differentially expressed genes between parental and prexasertib-resistant ovarian tumor cell lines. See Supplementary Table 11

Supplementary Table 12: Expression of immunity genes in tumor models with acquired or intrinsic resistance to prexasertib. See Supplementary Table 12

Supplementary Table 13: Short-hairpin genomic screen for sensitizers in prexasertib-resistant NCI-H520 tumor line. See Supplementary Table 13

Supplementary Table 14: Short-hairpin genomic screen for de-sensitizers in parental NCI-H520 tumor line. See Supplementary Table 14

Supplementary Table 15: CRISPR genomic screen for de-sensitizers in OV90 ovarian tumor line. See Supplementary Table 15

Supplementary Table 16: List of tumor cell lines used in the current study

Name	Histology	Source
NCI-H520	Squamous Lung	ATCC
SJC-Rh30	Alveolar Rhabdomyosarcoma	St Jude
SJC-Rh41	Alveolar Rhabdomyosarcoma	St Jude
OV90	Ovarian	ATCC
OVSAHO	Ovarian	ATCC
EFO21	Ovarian	ATCC
OVCAR-3	Ovarian	ATCC
KURAMOCHI	Ovarian	ATCC
MDA-MB-468	Breast	ATCC
MDA-MB-231	Breast	ATCC

Supplementary Table 17: List of antibodies used in the current study

Antibody	Vendor	Cat #	Species	MW (Kda)	Dilution
ATM	Cell Signaling	2873	Rabbit	350	1:1000
ATR	Cell Signaling	2790	Rabbit	250	1:500
53BP1	Abcam	ab21083	Rabbit	220	1:1000
BRCA1	Cell Signaling	14823	Rabbit	207	1:1000
Topo II a	Abcam	ab52934	Rabbit	174	1:2000
pMCM2 (S40/41)	Bethyl	A300-788A	Rabbit	125	1:1500
FANCD2	Santa Cruz	sc-20022	Mouse	150	1:1000
pRB	BD	558385	Mouse	110	1:1000
E-Cadherin	Millipore	07-697	Rabbit	106	1:1000
Wee1	Cell Signaling	13084	Rabbit	95	1:1000
FBXW7	Abcam	ab171961	Rabbit	79	1:1000
Mre11	Abcam	ab214	Mouse	75	1:1000
p-CDC25A(S76)	MyBiosource	MBS128525	Rabbit	70	1:1000
p-CDC25A(S178)	MyBiosource	MBS9200987	Rabbit	70	1:1000
cdc25A	Santa Cruz	SC7389	Mouse	67	1:500
pCHK2 (T68)	Cell Signaling	2661	Rabbit	62	1:500
p-cdc25c(S216)	Cell Signaling	4901	Rabbit	60	1:1000
cdc45	Santa Cruz	SC20685	Rabbit	60	1:1000
Myt1	Cell Signaling	4282	Rabbit	60 to 70	1:1000
pChk1 (S296)	Cell Signaling	90178	Rabbit	56	1:1000
PChk1 (S317)	Cell Signaling	2344	Rabbit	56	1:1000
pChk1 (S345)	Cell Signaling	2348	Rabbit	56	1:1000
Mus 81	Abcam	ab14387	Rabbit	65	1:1000
p-Chk2(Thr68)	Cell Signaling	2661	Rabbit	62	1:1000
p-Chk2(S516)	Cell Signaling	2669	Rabbit	62	1:1000
Cyclin B1	Cell Signaling	4135	Mouse	55	1:1000
Chk1	Santa Cruz	SC8408	Mouse	56	1:500
Chk2	Millipore	05-649	Mouse	56	1:500
Cyclin E	Abcam	ab33911	Rabbit	48-56	1:1000
p-MEK1/2(Ser217/221)	Cell Signaling	9154	Rabbit	45	1:1000
phospho-MAPK(ERK1/2)(Thr202/ Tyr204)	Cell Signaling	4376	Rabbit	42,44	1:2000
SKP2	Cell Signaling	2652	Rabbit	48	1:1000
pNPM(T199)	Cell Signaling	3541	Rabbit	38	1:1000
cdk2	Millipore	05-596	Mouse	33	1:1000
p-CDK2(Y15)	Abcam	ab76146	Rabbit	34	1:1000
p-cdc2(Tyr15)	Cell Signaling	9111	Rabbit	34	1:1000
pCDK2(T160)	Cell Signaling	2561	Rabbit	33	1:1000
pRPA32 (S4/S8)	Bethyl	A300-245A	Rabbit	32	1:1000
pRPA32 (T21)	Abcam	ab109394	Rabbit	32	1:1000
pRPA32 (S33)	Bethyl	A300-246A-T	Rabbit	32	1:1000
RPA32	Cell Signaling	2208	Rat	32	1:1000
HAUS1	Thermo	MA5-22937	Mouse	32	1:1000
p27	Cell Signaling	2552	Rabbit	27	1:1000
pp27(T187)	Abcam	ab75908	Rabbit	27	1:500
p21	Cell Signaling	2947	Rabbit	21	1:2000
gH2AX	Millipore	05-636	Mouse	17	1:5000
